# Correction: Vaccine equity in low and middle income countries: a systematic review and meta-analysis

**DOI:** 10.1186/s12939-022-01695-4

**Published:** 2022-07-07

**Authors:** Huda Ahmed Ali, Anna-Maria Hartner, Susy Echeverria-Londono, Jeremy Roth, Xiang Li, Kaja Abbas, Allison Portnoy, Emilia Vynnycky, Kim Woodruff, Neil M. Ferguson, Jaspreet Toor, Katy A. M. Gaythorpe

**Affiliations:** 1 Imperial College London, Praed Street, London, UK; 2 London School of Hygiene and Tropical Medicine, Keppel Street, London, UK; 3 Center for Health Decision Science, Harvard T H Chan School of Public Health, Cambridge, USA; 4 Public Health England, London, UK

**Correction: Int J Equity Health 21, 82 (2022)**

**https://doi.org/10.1186/s12939-022-01678-5**

After publication of this article [1], the authors reported that

(1) In the Results section of the Abstract the penultimate sentence should have read “Additionally, we estimated that children whose mothers had no formal education were 27% (95%CI[16%,36%]) less likely to be fully vaccinated than those whose mother had primary level, or above, education.” instead of “Additionally, we estimated that children whose mothers had no formal education were 28% (95%CI[18%,47%]) less likely to be fully vaccinated than those whose mother had primary level, or above, education.”

(2) In the fourth paragraph of the Discussion section, the sentence “We estimated that children were 27% (95%CI [16%,36%]) more likely to be fully vaccinated if their mother had primary level education or above, compared to no education.” should have read “We estimated that children were 27% (95%CI [16%,36%]) less likely to be fully vaccinated if their mother had no formal education, compared to primary education or above.”

The original article [1] has been updated.
